# Pertussis outbreak investigation in Northwest Ethiopia: A community based study

**DOI:** 10.1371/journal.pone.0263708

**Published:** 2022-02-10

**Authors:** Addisu Gize Yeshanew, Damtie Lankir, Jimmawork Wondimu, Samrawit Solomon

**Affiliations:** 1 Depatment of Microbiology, St. Paul’s Hospital Millennium Medical College, Addis Ababa, Ethiopia; 2 Amhara Public Health Institute, Public Health Emergency Management (PHEM) directorate, Bahir Dar, Northwest Ethiopia; 3 Depatment of Public Health, St. Paul’s Hospital Millennium Medical College, Addis Ababa, Ethiopia; Public Health England, UNITED KINGDOM

## Abstract

**Background:**

Pertussis or whooping cough is a vaccine-preventable, highly contagious, respiratory illness caused by *Bordetella pertussis* or *Bordetella parapertussis*. Infants and young children have remained most susceptible to pertussis-related morbidity and mortality. The aim of this study was to investigate pertussis infection and analyze the associated factors involved in the occurrence of the cases.

**Methods:**

Community-based case-control was conducted in Dahena district, Northwest Ethiopia, from March 27—April 30, 2019. All cases ages 1–18 years old were identified by using the clinical standard case definition of pertussis adopted from World Health Organization (WHO). Data was collected using a structured questionnaire via face-to-face interviews. The data collected was cleaned, coded and entered into Epi info version 7.2.1.0 and exported to SPSS version 23 for statistical analysis. Bivariable and multivariable logistic regression analysis were employed to identify predictors. Factors with a p-value of < 0.05 were considered as independent risk factors of pertussis infection in multivariable logistic regression analysis.

**Result:**

A total of 122 pertussis cases were enrolled from the Azila cluster of the Dahena district. Of these figures, 64 (52.5%) were females. The overall attack rate (AR) of pertussis cases in the cluster was 8.6/10000 population. The sex-specific AR of females was 8.9/10000 population. The multivariable logistic regression analysis showed that; being unvaccinated 4.17 (AOR, 4.17, 95% CI, 1.914–9.091), contact to cases 2.93 (AOR: 2.93, 95% CI 1.223–6.996), and living in a house with no window 2.6 (AOR: 2.6(95% CI 1.071 to 6.322) were the independent significantly risk factors for pertussis infection.

**Conclusion:**

The contributing factor for pertussis infection was associated with case-contact, living in the house without windows and being unvaccinated. Wag Hemra Zone and Dahena district health office should encourage the vaccination activities of the cluster health center and awareness for the community should be practiced to limit disease transmission.

## Background

Pertussis or whooping cough is vaccine-preventable [1], Gram-negative, pleomorphic aerobic coccobacillus, and highly contagious respiratory illness caused by *Bordetella Pertussis* or *Bordetella Para Pertussis* [2]. It grows optimally on either Bordet-Gengou or Regan-Lowe agar between 35°C and 37°C and it is also a fastidious, non-motile, catalase, and oxidase-positive species [3].

Pertussis typically has three stages of symptoms. The first stage of the disease is the catarrhal stage; which is associated with sneezing, conjunctival suffusion, rhinorrhea or nasal congestion, low-grade fever (minimal), and a mild, occasional cough. The second stage is the paroxysmal stage; characterized by bursts of rapid coughs followed by a long inspiratory whoop and patients often become cyanotic during these episodes. Cough is particularly severe at night and frequently followed by post-tussive vomiting (emesis) in typical cases. This stage usually lasts for 1–6 weeks but may persist for up to 10 weeks. The last stage is the convalescent stage, in which recovery is gradual. The cough becomes less paroxysmal and usually disappears within 2 to 3 weeks. However, paroxysms may recur and this stage may last for several months [4,5].

Pertussis transmission occurs via the respiratory route to others by direct, close contact with secretions from the nose, throat, and mouth of an infected person. A sneeze or droplets from a cough can spread the disease to others. A person with pertussis may be contagious for as long as 3 weeks after and 2 weeks before symptoms begin [6]. Humans are the only known reservoir for pertussis; no animal or insect source or vector is known to exist [7,8].

Living in the same house with an infected person, waning immunity following vaccination, and not being immunized are major risk factors for the occurrence of pertussis cases and outbreaks [8,9]. People in the same household who have not had their Diphtheria Pertussis and Tetanus (DPT) vaccines as infant and adults are 80 to 100 percent likely to be infected with exposure, but those who have been immunized and live in the same household, are 20 percent likely to be infected [9].

*Bordetella pertussis* is life-threatening in infants [10]. Secondary bacterial pneumonia is the most common complication and cause of most pertussis-related death. Pertussis-associated complications are highest in young infants. Adolescents and adults are often the source of infection for children and an important reservoir for *B*. *pertussis* [9]. Lymphocytosis is useful and a major diagnostic tool for pertussis infection in young children and infants [10].

Infants and young children have remained most susceptible to pertussis-related morbidity and mortality [11]. The highest risk of severe complications (including pneumonia, apnea, and seizures) is common to young unvaccinated infants. In recent years infants younger than 6 months who are not old enough to have received three doses of DPT vaccine and under-vaccinated preschool children have been at higher risk for pertussis-associated complications [12].

Globally millions of cases and tens of thousands of deaths occur annually due to pertussis infection, as the study suggested worldwide, 48.5 million cases of children are affected by the disease. Of this figure, younger than four years and five to fourteen years old age groups were found to be affected [13].

In 2014, out of 24·1 million pertussis cases, 160,700 deaths worldwide, are attributed to children younger than 5 years, the African region contributed 92,500 (58%) of deaths and 7.8 million (33%) cases. The estimated cases and deaths in younger than 1 year were 5.1 million (21%) and 85,900 (53%) respectively [14].

The magnitude of the disease is more and more severe in developing countries, or in the African continent because of poor disease management, detection, and lack of awareness. In East African countries, the study conducted in Kenya revealed that before and after vaccination the attack rate was highest among infants and younger children [15].

When we see the vaccination status related to the disease in the continent, DPT3 coverage varied in the district areas of most African countries by 25%, excluding Morocco and Rwanda (which met the global vaccine action plan). In 2016, Somalia, Angola, and Ethiopia were listed among countries which have low DPT3 coverage, which was ≤50% and more than ≥30% dropout [16]. Infant mortality rate of the disease is high, even in countries which have routine vaccination schedules like in South Africa, and adults who have HIV-infection identified as risk factors for *B*. *pertussis* infection [17].

The southern part of our country indicated that the overall attack rate was 1708 per100,000 population, and the fatality rate 3.3 per 1000 cases. This attack rate was very high in infant age groups and only 41% had completed the three-dose pertussis vaccine primary schedule, whereas the household survey resulted in 73% pentavalent vaccine coverage [18].

Regarding the region, Amhara Public Health Institute (APHI), Public Health Emergency Management (PHEM) directorate seven-month summary report, from July 1, 2018, to January 30, 2019, a total of 1620 registered pertussis cases were reported from different districts [19].

The case fatality rate of the disease near the study area showed 3.3%. According to this specific area study, gender, contact with suspected persons, and housing without ventilation are considered as risk factors for the pertussis disease [20]. Another study revealed the overall attack rate of the disease 1.3 per 1000 population, and previous history of sickness with the disease and receiving full dose of the vaccine remained as protective from the disease [21]. The assessment of the vaccine efficacy study showed 31% infection among the vaccinated groups. The vaccine protectiveness for the participants who had complete vaccination also showed 50% having a little age difference. Even, the availability and good coverage of childhood vaccination, its effectiveness was found to be low in the Amhara region, Ethiopia [22], and the pentavalent dropout vaccination rate from a three-dose to single-dose in Dahena district was 11.3% [19].

Therefore, it is reasonable to investigate pertussis outbreak, describe risk factors and proposed appropriate intervention measures, Dahena district, Wag Hemra Zone, Northwest Ethiopia.

## Methods

### Study setting

Information about the cases were obtained from the Amhara public health institution, Northwest Ethiopia, and investigators were assigned for field work at the site. They confirmed an outbreak in the Azila cluster of Gomengie and Seramre Kebeles of Dahena district. Dahena is one of the districts in the Wag Hemra Zone Amhara region, Northwest Ethiopia. The district is bordered on the South by the North Wollo Zone (Bugna district), on the West by South Gondar Zone (Ebinat district), on the North by Zikuala, on the northeast by Sekota, and on the East by Gazgibla districts. It has a latitude and longitude of 12° 25’ 42.1608’’ N, 38° 42’ 57.0348’’ E. The district is located 487 kilometers (km) far from Bahirdar and 78 km from the zonal town Sekota. The major town in Dahena is Amdework. In this district, there are six clusters with six-health centers and 31 health posts. Azila is one of the clusters from the district with a total population of 17236. This cluster had three kebeles named as Gomengie, Seramre, and Tilala. From the three kebeles in the cluster, Gomengie and Seramre kebeles were on an outbreak of pertussis and selected for investigation. Kebele is the smallest administrative unit of Ethiopia, and a part of a district.

### Study design and period

Unmatched community-based case-control was conducted in the Azila cluster of Seramre and Gomengie kebeles of Dahena district from March 27- April 30, 2019 in order to analyze the associated factors involved in the occurrence of the cases and to propose possible control measures.

### Source and study population

The source and study population were all suspected pertussis cases in the community and who had a history of coughing for more than two weeks in Gomengie and Seramre kebeles of the Dahena district respectively.

### Enrolment of cases and controls

A suspected case definition adopted from the World Health Organization (WHO) was used. A case was defined as experiencing a non-improving cough illness greater or equals to two weeks with at least one of the following signs or symptoms: Paroxysms of coughing or, inspiratory whoop or, post-tussive vomiting, or apnea between March 18, 2019 to April 30, 2019. A control was defined as any person without showing any signs and symptoms of pertussis during outbreak investigation from March 18 to April 30, 2019, in the Dahena district within the study period.

### Inclusion and exclusion criteria

All pertussis cases in Gomengie and Seramre Kebeles who had a history of cough for more than two weeks for cases and the history of known vaccination for cases and control were included. Controls that had a history of cough for at least one day before data collection and unknown history on vaccination for cases, contact with a suspected case in the case of control groups, were excluded from the study.

### Sampling and Sample size determination

The total sample size was 182 (61 cases and 121 controls in the ratio of one to two). The sample size was determined by Fleiss formula using the Epi Info Version 7.2.1.0 software by considering one variable assumed to bring a difference in the two groups. The sample size calculation was based on two-sided CI = 95%, Power = 80%. From a similar study conducted in the Mekdela district, receiving full dose vaccination was taken as the main predictor of the outcome (pertussis infection). In that study, the percent of controls exposed is 27% with OR of 0.256 [21]. Cases were selected using a simple random sampling technique from all recorded cases up to the recommended sample size reached. Two controls were selected for each enrolled pertussis case who were present during the visited date based on the fulfillment criteria.

### Data collection instrument

A structured questionnaire and a face-to-face interview were used to collect data. A developed collection tool was adopted from the WHO surveillance assessment tool and from previously published similar study [21]. Data collection tool contains socio-demographic variables, clinical manifestation and complications, risk factors and immunization status.

### Data collection procedure

To collect socio demographic, immunization status, and possible factors of pertussis infection cases and controls were interviewed using structured questionnaires. Active cases search was done through house-to-house visits. Cases were taken and followed daily within the study period. All information hypothesized as risk factors for the pertussis outbreak were collected.

### Data quality control

Members of the team were discussed on the questionnaires in detail to have a common understanding of the tool. The collected data was cross-checked daily through recorded cases before and during data entry and processing for its completeness and consistency in the study period.

### Data processing and analysis

Epi info version 7.2.1.0 was used for data entry and edition, and then it was exported into SPSS version 23 for data analysis. Descriptive statistics were used to present socio-demographic variables. The strength of association between pertussis cases and independent variables (covariates) was expressed as an OR with a 95% confidence interval. Binary logistic regression analysis was done and all variables that were found to be significant at p-value <0.2. Independent variables that were at p-value <0.05 levels in the multivariable logistic regression analysis were considered as significant factors for pertussis infection.

### Ethical issues

Permission letter was obtained from Amhara public health institute public health emergency management directorate which was located in Amhara region and stated the ethics number as (APHI/PHEM/5.05/6285, 19/07/2011). The purposes and the importance of the study were stated to Wag Hemra Zone health department, Dahena district health office, and to the cluster health center for their co-operation and facilitating the outbreak investigation. The purposes of the outbreak investigation were explained to respondents and verbal consent was obtained from a parent or guardian for participants under 16 years old before interviewing and agreeing to take part in the investigation. Confidentiality was assured at all levels of the study using password protected computers and through deleting all identifiers.

### Operational definitions

**A suspected case of pertussis**: cough of 14 days or more or cough of any duration with paroxysms or cough of any duration with a whoop.

**Confirmed case of pertussis:** A case that meets the clinical case definition and is epidemiologically linked directly to a lab-confirmed case.

**Pertussis outbreak case definition**: two or more cases have occurred within 42 days of each other and clustered in a common setting (time and space) and one or more cases can be confirmed to be pertussis by positive culture results.

**Vaccinated**: an individual who had received at least one dose of pertussis-containing vaccine. Being vaccinated was verified by vaccination card or parents confirmed in an interview that she or he has received at least one pertussis vaccination.

**Unvaccinated**: an individual who had no vaccination card and whose parents or guardians confirmed in an interview that she or he has received no pertussis vaccination.

**Unknown vaccination**: an individual who had no vaccination card and whose parents or guardians cannot confirm in an interview that she or he has received pertussis vaccination.

## Results

### Socio-demographic distribution of pertussis cases

**Fig 1:**
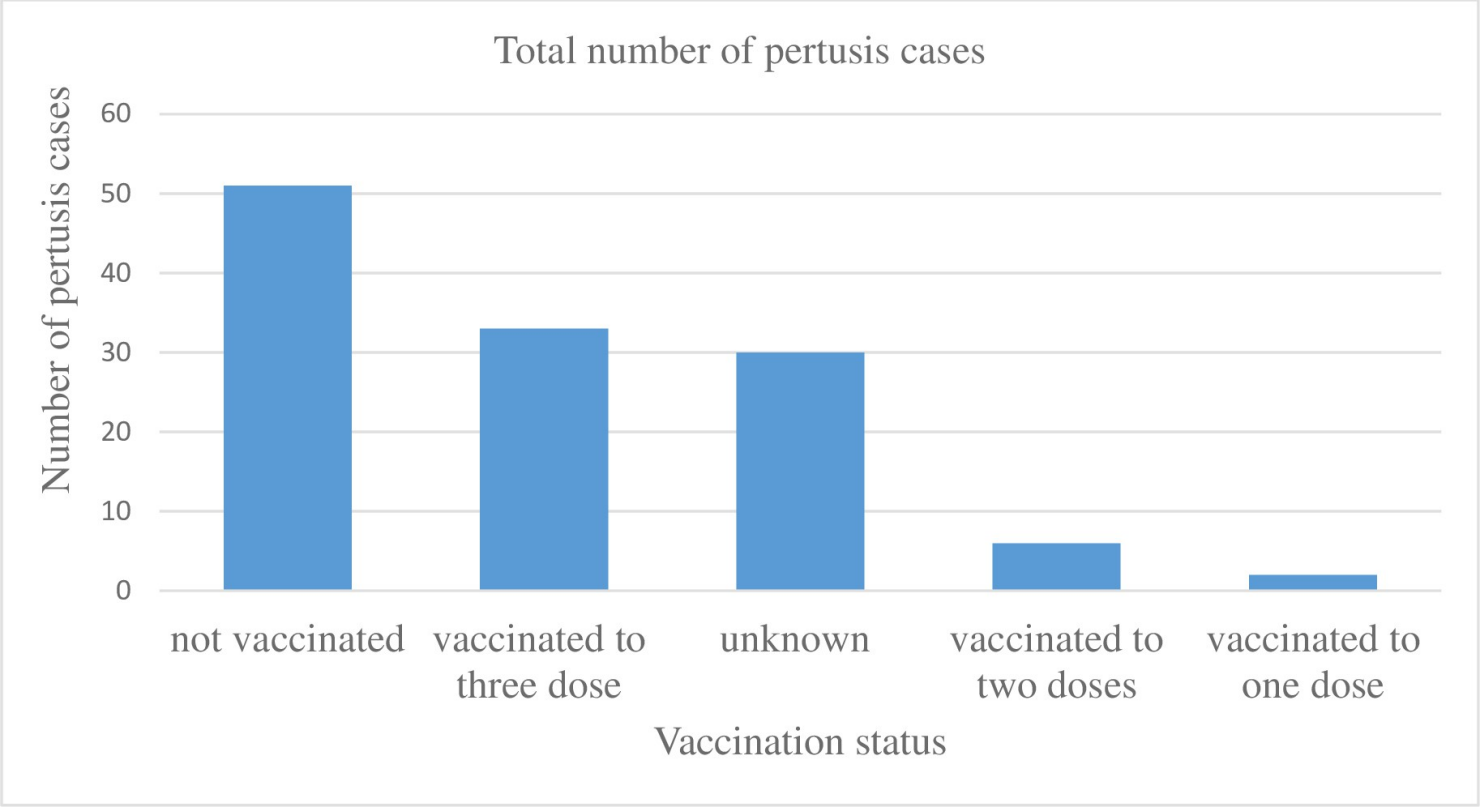
Vaccination status and pertussis cases in Gomengie and Seramre Kebele of Dahena district, 2019.

A total of 122 pertussis cases were reported from the Azila cluster of (Gomengie, 98 and Seramre, 24 cases) Dahena district. The median age of cases was 9 years (range from one to eighteen years). Sixty-four (52.5%) cases were females. Age group 5–9 years accounts for 51 (41.8%) cases followed by 1–4 years of age 32 (26.2%). From the total cases, 109 (90%) cases had a paroxysmal cough and whooping respectively. Thirteen (10.7%) of cases had post tussive vomiting. From all reported cases only four (3.3%) cases developed severe pneumonia, subconjunctival hemorrhage and had weight loss. Hernia and hospitalization was not reported due to these cases. The overall attack rate of cases in Gomengie and Seramre kebeles was 8.6/10000 population. The sex-specific attack rate of females was 8.9/10000 populations. The highest age-specific attack rate was between 5–9 years of age (24.5 cases per 10000 population). No deaths were reported in either Kebeles of the district. Sex and Age distribution of pertussis cases by kebeles with attack rate was presented in Table 1 and the vaccination status in relation to age categories in Table 2, whereas the vaccination status of cases to three dose, two dose, and not vaccinated were 33 (27.1%), six (4.9%) and 51 (41.8%) respectively, Fig 1.

**Table 1:** Attack rate of pertussis cases in Gomengie and Seramre Kebeles of Dahena district, April 2019.

Variables	Total population	Kebeles				Total cases (AR/10000)
		Gomengie		Seramre		
		Number of cases	AR /10000	Number of cases	AR/10000	
Sex						
Female	71,825	50	6.96	14	1.95	64 (8.9)
Male	69,388	48	6.92	10	1.44	58 (8.4)
Age group						
1–4	19163	24	19.44	10	6.77	34 (26.3)
5–9	20828	44	21.13	7	3.36	51 (24.5)
10–14	20166	19	9.42	5	2.48	24 (11.9)
15–18	81056	11	1.36	2	0.25	13 (1.6)
Total	141213	98	6.94	24	1.70	122 (8.6)

**Table 2:** Vaccination status of pertussis cases in the Azila cluster of Dahena district, Wag Hemra Zone, Amhara region Ethiopia, 2019.

Sex and Age categories	Vaccination status					
	Un vaccinated	%	Vaccinated to Penta-3	%	unknown	%
Sex						
Female	25	20.5	25	20.5	14	11.5
Male	26	21.3	16	13.1	16	13.1
Ages group						
1–4	3	2.5	28	23	1	0.8
5–9	24	19.7	12	9.8	15	12.3
10–14	14	11.5	0	0	10	8.2
≥15	10	8.2	0	0	3	2.5
Total	51	41.8	41	33.6	30	24.6

### EPI-curve of pertussis cases by date of onset of disease

**Fig 2:**
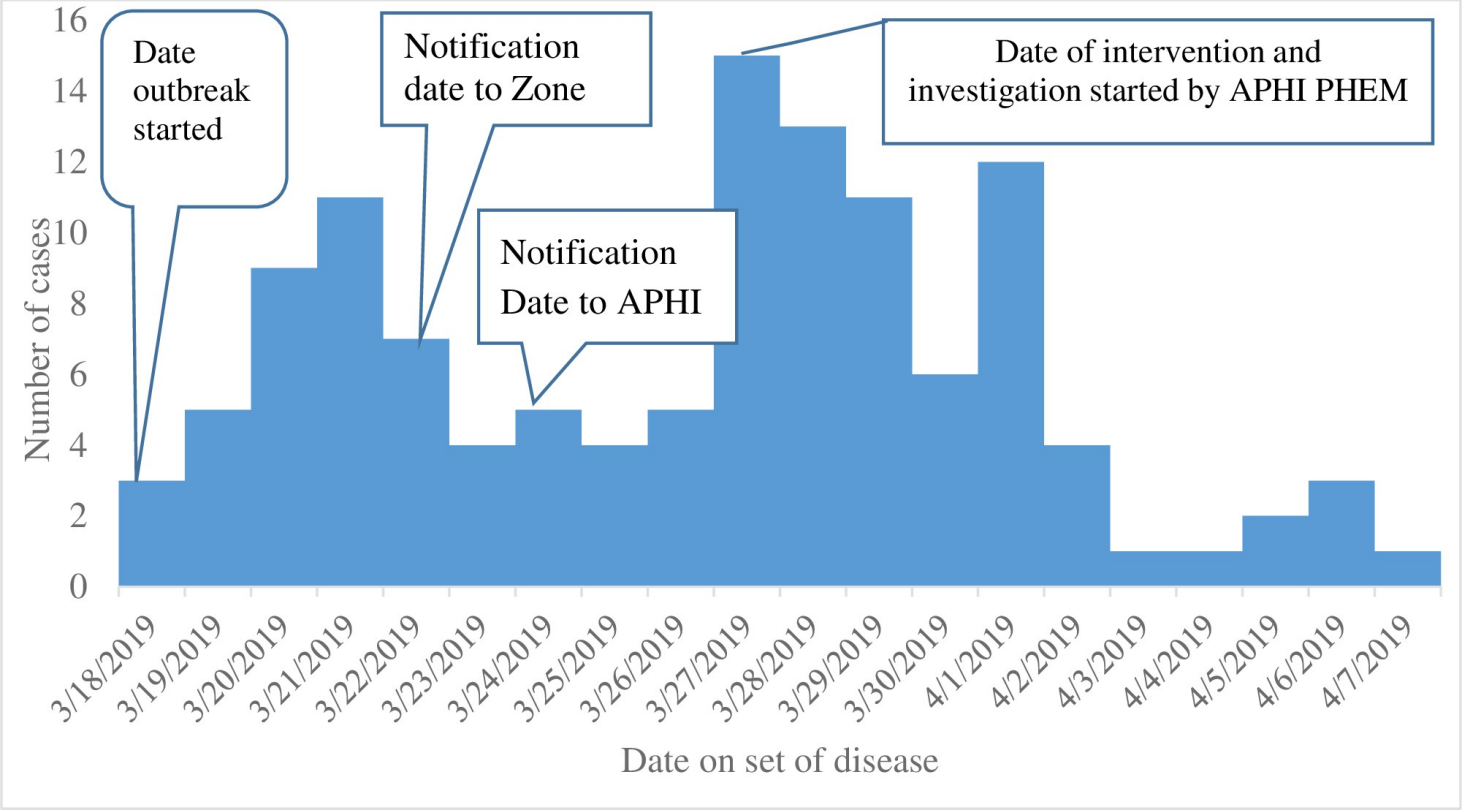
EPI curve of the pertussis outbreak over time in Gomengie and Seramre Kebele, April 2019. APHI = Amhara Public Health Institute.

A propagated type epidemic, it begun with three cases and raised gradually by the number of cases (upslope curve) and a 21 days duration of pertussis outbreak occurred in the Azila cluster of Dahena district. After seven days of outbreak started, the Wag Hemra Zone health department notified the occurrence of pertussis outbreak to the APHI PHEM directorate on March 24, 2019, on a weekly based report. The outbreak was started on March 18, 2019 and intervention was begun on March 27, 2019. The date onset of disease and the number of reported cases with a date of investigation and intervention started by APHI PHEM and the district health office were presented in Fig 2.

### Analytical investigation

A total of 61 cases and 121 controls residing in the Azila cluster were selected for case-control study with a ratio of one to two respectively. From total study subject 29 (47.5%) cases and 29 (24%) controls had no prior to any dose of vaccination. Forty-seven (77%) cases and 81(67%) controls do not know the mode of pertussis transmission. Of all participants, 132 (72.5%) had a history of contact with a case-patient and 98 (53.9%) had no information about the pertussis vaccine. Regarding housing conditions, 52 (85.2%) cases and 87 (71.9%) controls were lived in the houses without windows, Table 3.

**Table 3:** Characteristics of pertussis cases and controls in Azila cluster of Dahena district, April 2019.

Characteristics	Cases		Controls		Total (%)
	N = 61	%	N = 121	%	N (%) = 182 (100)
Sex					
Male	21	34.4	52	43	73 (40.1)
Female	40	65.6	69	57	109 (59.9)
Age categories					
1–4	20	32.8	48	39.7	68 (37.3)
5–9	25	41.0	35	28.9	60 (33)
10–14	11	18.0	30	24.8	41 (22.5)
15–18	5	8.2	8	6.6	13 (7.1)
Marital status of mother/ caregivers					
Unmarried	9	14.8	22	18.2	31 (17)
Married	52	85.2	99	81.8	151 (83)
Mother’s /caregivers occupation					
House wife	44	72.1	90	74.4	134 (73.6)
Farmer	17	27.9	31	25.6	48 (26.4)
The education level of mothers /caregivers					
Unable to read and write	49	90.2	96	79.3	145 (79.7)
Primary	12	9.8	25	20.7	37 (20.3)
Family size					
≥ 7	12	19.7	27	22.3	39 (21.4)
5–6	26	42.6	42	34.7	68 (37.4)
1–4	23	37.7	52	43	66 (41.2)
Vaccinated status					
Unvaccinated	29	47.5	29	24	58 (31.9)
Vaccinated to Penta-1	9	14.8	12	9.9	21 (11.5)
Vaccinated to Penta-2	6	9.8	14	11	20 (11)
Vaccinated to Penta-3	17	27.9	66	54.5	83 (45.6)
History of contact with cases					
Yes	53	86.9	79	65.3	132 (72.5)
No	8	13.1	42	34.7	50 (27.5)
Know the method of pertussis transmission					
No	47	77	81	67	128 (70.3)
Yes	14	23	40	33	54 (29.7)
Know the method of pertussis prevention					
No	41	67.2	74	61.2	115 (63.2)
Yes	20	32.8	47	38.8	67 (36.8)
Know pertussis is vaccine preventable disease					
No	40	54.1	58	55.4	98 (53.9)
Yes	21	45.9	63	44.6	84 (46.1)
Place of residence from vaccination site					
> 5 km	24	39.3	70	57.9	94 (51.6)
≤ 5 km	37	60.7	51	42.1	88 (48.4)
Housing condition					
No window	52	85.2	87	71.9	139 (76.4)
≥ One windows	9	14.8	34	28.1	43 (23.6)

### The bi-variable and multivariable logistic regression analysis result

Socio-demographic, vaccination status, and other risk factors were analyzed in the bivariable logistic regression to identify risk of pertussis infection. In bivariable logistic regression analysis, there were no differences between cases and controls by gender (p = 0.27), age categories (p > 0.5), family size (p > 0.05) and level of education of caregivers (p = 0.88). Marital status, education and occupation of the mother/caregiver, and family size were found also not to make a difference in the case vs control group. However, the history of receiving three doses of vaccine, history of contact to cases (with diseased), known pertussis is vaccine preventable disease, place of residence from vaccination site and housing condition were different between cases and control. In multivariable logistic regression analysis three variables showed a significant association. Being unvaccinated is 4.17 times more risk to develop pertussis infection than vaccinated AOR: 4.17 (95% CI 1.91 to 9.09). A person having contact to cases was 2.93 more likely to develop pertussis infection than having no contact to cases AOR: 2.93(95% CI 1.22 to 6.99). Living in the house without a window was 2.6 more likely to develop pertussis infection than a person living within the house of window AOR: 2.6 (95% CI 1.07 to 6.32), Table 4.

**Table 4:** Bivariable and multivariable logistic regression analysis of risk factor for pertussis infection in the Azila cluster of Dahena district, 2019.

Variables	Case N (%)	Control N (%)	Bivariable Analysis		Multivariable Analysis	
			COR (95% CI)	p-value	AOR (95% CI)	p-value
Sex						
Male	21 (34.4)	52 (43)	0.697 (0.368–1.320)	0.268		
Female	40 (65.6)	69 (57)	1			
Age categories						
1–4	20 (32.8)	48 (39.7)	1.159 (0.202–2.445)	0.605		
5–9	25 (41)	35 (28.9)	1.143 (0.334–3.908)	0.831		
10–14	11 (18)	30 (24.8)	0.587 (0.158–2.182)	0.426		
15–18	5 (8.2)	8 (6.6)	1			
Marital status of mothers/ caregivers						
Married	52 (85.2)	99 (81.8)	1.284 (0.552–2.989)	0.562		
Unmarried	9 (14.8)	22 (18.2)	1			
Mother’s/caregiver’s occupation						
House wife	44 (72.1)	90 (74.4)	0.892 (0.446–1.782)	0.745		
Farmer	17 (27.9)	31 (25.6)	1			
Mothers/caregivers education level						
Unable to read and write	49 (80.3)	96 (79.3)	1.063 (0.493–2.296)	0.876		
Primary	12 (19.7)	25 (20.7)	1			
Family size						
≥ 7	12 (19.7)	27 (22.3)	1.005 (0.434–2.324)	0.991		
5–6	26 (42.6)	42 (34.7)	1.400 (0.700–2.978)	0.342		
1–4	23 (37.7)	52 (43)	1			
Vaccination status						
Unvaccinated	29 (47.5)	29 (24)	3.882 (1.850–8.145)	< 0.001*	4.172 (1.914–9.09)	<0.001**
Vaccinated to Penta-1	9 (14.8)	12 (9.9)	2.912 (1.055–8.038)	0.039*	3.91 (1.295–11.81)	0.016**
Vaccinated to Penta-2	6 (9.8)	14 (11.6)	1.664 (0.557–4.973)	0.362	1.57 (0.511–4.833)	0.431
Vaccinated to Penta-3	17 (27.9)	66 (54.5)	1		1	
History of contact with a case						
Yes	53 (86.9	79 (65.3)	3.522 (1.532–8.096)	0.003*	2.925 (1.223–6.996)	0.016**
No	8 (13.1	42 (34.7)	1		1	
Know the way of pertussis transmission						
No	47 (77)	81 (67)	1.658 (0.818–3.361)	0.161*	1.29 (0.574–2.900)	0.538
Yes	14 (23)	40 (33)	1			
Know the methods of pertussis prevention						
No	41 (67.2)	74 (61.2)	1.302 (0.681–2.488)	0.424		
Yes	20 (32.8)	47 (38.8)	1			
know pertussis is vaccine preventable disease						
No	40 (65.6)	58 (47.9)	2.069 (1.094–3.913)	0.025*	1.126 (0.417–3.042)	0.815
Yes	21 (34.4)	63 (52.1)	1		1	
Place of residency from vaccination site						
> 5 km	24 (39.3)	70 (57.9)	0.473 (0.252–0.885)	0.019*	1.372 (0.516–3.647)	0.526
≤ 5 km	37 (60.7)	51 (42.1)	1		1	
Presence of window in the house						
No	52 (85.2	87 (72)	2.258 (1.003–5.081)	0.049*	2.602 (1.071–6.322)	0.035**
Yes	9 (14.8	34 (16)	1		1	

* Nominated variables for multivariable logistic regression analysis.

1 = Reference category,

** indicates statistically significant at p-value of < 0.05.

## Discussion

From 122 pertussis cases reported, the AR of the 5–9 age group were 24.5/10000 population which was highly affected as compared to other age groups. The AR of age group 1–4 and 10–14 years were 21.7/10000 population and 11.9/10000 population respectively. The overall attack rate of pertussis cases in the Azila cluster was 8.6/ 10000 population. This result is different from a study conducted in South Wollo Zone of Mekdela district, which showed that from July to October 2015, out of 215 pertussis cases most affected age group was under 4 years 123 (57.2%) with the AR of 6.8 per 1000 population and overall AR of 1.3 per 1000 inhabitant [21]. This result variation may be due to geographical location, vaccination difference of the district and study year variation between the two districts.

In this study, 51 (41.8%) cases had no vaccination history. Thirty-three (27.1%) cases received three doses of pertussis vaccine. This finding is different from a report of the Amhara region 2018 annual vaccination coverage of the district, which was Penta-1 and Penta-3 vaccination coverage of the Dahena district were 115% and 102% respectively with a Penta-one to three dropout rate of 11.3% [19]. Even though vaccination coverage of the affected district was greater than 100% in 2018, pertussis outbreak was the problem in the district; this might be due to problem of reporting fallacy or double reporting of the district to region, poor potency of the vaccine, and utilization problem of the parents in the district.

Females 64/122 (52.5%) were more affected than males. The most affected age groups were those aged 5–9 (41.8%) followed by 1–4 (26.2%) and 10–14 (19.7%). The result of this outbreak investigation was different from the outbreak investigation of New Brunswick 2014, which shows, cases were highest in 10–14 years age group (38%) followed by 20 years (28%), 5–9 year (15%) and 15–19 and 1–4 year (8%) [23]. This variation might be due to vaccination coverage differences in the country and it needs further investigation.

In this outbreak investigation, participants who did not know the mode of transmission and prevention of pertussis were 128 (70.3%) and 115(63.2%) respectively. The exposure status of the participant to cases was 132 (72.5%). Regarding housing conditions, 139 (76.4%) of the participants lived in a house with one room without a window. The likelihood-contributing factor might be due to poor seeking behavior of people, low educational status and their living environment difficulty.

This outbreak investigation identified the factor that the history of contact to cases was 2.925 times more at risk to develop pertussis infection than those who have no contact to cases. This remained significantly associated with the occurrence of a pertussis infection in Gomengie and Seramre kebeles of the Dahena district. This study result is similar to a study conducted in the South Wollo Zone of Mekdela district [23]. This might be due to similar living standards, vaccination habits of the parents, and family size similarity of the Mekdela and Dahena districts. This study is also aligned with a study conducted in the Republic of Korea [8] and Northern Nigeria [24]. This might be due to inter-relation of family members increasing the frequency of contacts to cases even though they were living in different standards, geographical conditions, and different strategies used to address immunization to prevent pertussis transmission.

In this outbreak investigation, children who received a full dose of pertussis vaccine were less likely to develop pertussis infection. Being unvaccinated is 4.172 times more risk to develop pertussis infection AOR: 4.172 (95% CI 1.914 to 9.091). This might be due to respondents having no knowledge about pertussis as Vaccine-Preventable Disease (VPD), inaccessible to the vaccination site, poor knowledge of respondents about the mode of prevention of Pertussis infection in the district.

Mortality was not reported due to pertussis infection in the Dahana district. This study is different from a study conducted in the South Wollo Zone of Mekdela district [21]. This study variation might be due to immediate outbreak investigation and early treatment of cases in the Dahena district, as we assured in our study that the outbreak was abated and stopped after April 2019 due to possible interventions.

### Limitations of the study

Confirmed and epidemiological linked cases of pertussis were not verified due to a lack of laboratory diagnostic methods at a national and regional level at the time of the outbreak. Absence of vaccination cards was difficult to determine the vaccination status, the exact date of vaccination and other relevant information that could cause information bias. Recall bias on the date of onset by the cases and their mothers occurred. Lack of literature related to pertussis outbreak investigation at national, regional and district levels causes referencing problems.

### Intervention measures of the outbreak

Case management: Antibiotics were given to all suspected pertussis cases to prevent complications and death in the affected Kebeles in collaboration with the district health office, which was the principal goal of the intervention.

Health information: health information was given on the importance of vaccination and the advantage of taking the diseased to the nearby health facility; the complication that may be resulted from the disease if someone is left untreated. The surveillance system of the district was also assessed, which was not well organized and the rapid response team of the district had no programmatic supervision to the cluster before the outbreak was raised. The Functional Refrigerator was not available for the storage of vaccines. Vaccines were transported by delegated focal persons of the health development army without proper management of vaccines.

Active case search: Despite the difficult landscape of the area, which was highly labor-intensive, home-to-home activities of case search were done to cover more cases.

## Conclusions

Most of the cases were preschool-age children who had contact history with pertussis cases at home or with neighbors and had received no prior dose of pertussis-containing vaccine. The disease of pertussis is associated with the case contact, unvaccinated and dwelling condition. Awareness creation for the community about the transmission, prevention and control mechanisms should be needed to limit the disease transmission when the cases present. Functional refrigerators should be available for the health posts and cluster health centers to maintain the potency of vaccines. Inhabitants of the community need to be mobilized to increase their awareness of the importance of immunization and health service seeking behavior. The cluster health center should have to establish and implement routine Expanded Program of Immunization (EPI) services near to Gomengie and Seramre Kebele of the Dahena district.

## Supporting information

S1 File (DOCX)

## Abbreviations

AOR: Adjusted Odds Ratio
APHI: Amhara Public Health Institute
AR: Attack Rate
B. pertussis: Bordetella pertussis
CFR: Case Fatality Rate
CI: Confidence Interval
DPT: Diphtheria Pertussis and Tetanus
EPI: Expanded Program of Immunization
EPI Curve: Epidemic Curve
Km: Kilometer
LR: Logistic Regression
PHD: Public Health Department
PCR: Polymerase Chain Reaction
PHEM: Public Health Emergency Management
SPHMMC: Saint Paul’s Hospital Millennium Medical College
SPSS: Statistical Package for Social Science
OR: Odds Ratio
WHO: World Health Organization
