# Supplementary material for: Pertussis outbreak investigation in Northwest Ethiopia: A community based study
Source: PLoS One. 2022 Feb 10;17(2):e0263708. doi: 10.1371/journal.pone.0263708 (PMC8830691; doi:10.1371/journal.pone.0263708)
Supplement: S1 File — (DOCX) [file pone.0263708.s001.docx]

**English Version questionnaires for pertussis outbreak investigation data collection**

Investigators: ____________________________Date ______________ID No_________

Respondent: Patient Family member (Mother, Father: Brother or sister or other relatives close to patient.)

Respondent’s status. 􀀀 Case 􀀀 Control

| 1. **Socio-demographic variables** | | | | | |
| --- | --- | --- | --- | --- | --- |
| Sr no | | Questions | Option/Response | | Remark |
| 1 | | Age | __________(in years) | |  |
| 2 | | Sex | 1. Female 2. Male | |  |
| 3 | | What is your religion? | 1. Orthodox 2. Protestant 3. Muslim 4. Others(specify)------------ | |  |
| 4 | | Occupation of cases/control? | 1. N/A 2. Farmer 3. Housewife 4. Student 5. Governmental Employee 6. Non- government employ 7. Others(specify)------------------- | |  |
| 5 | | Educational Status of Case/ control? | 1. N/A 2. Cannot read and write 3. Can read and write 4. Attend primary school (Grade 1-8) 5. Attend secondary school (Grade 9-12) 6. College diploma and above | |  |
| 6 | | Marital status of care giver | 1. Married 2. Single 3. Widowed 4. Separated 5. Divorced | |  |
| 7 | | Educational Status caregivers? | - - - 1. Cannot read and write       2. Can read and write       3. Attend primary school (Grade 1-8)       4. Attend secondary school (Grade 9-12)       5. College diploma and above | |  |
| 8 | | Occupation of caregivers? | Farmer  Housewife  Student  Governmental Employee  Non-government employ  Others(specify | |  |
| 9 | | Place of residence from vaccination site? | 1. $\leq$ five KM 2. > five KM | |  |
| 10 | | Total number of family member who lived in the house | _______________ | |  |
| 1. **Clinical manifestations and complication of cases** | | | | | |
| 11 | | Do you/ your child have any of the following clinical features  . | 1. Paroxysmal cough 2. Whooping 3. Post- tussive vomiting 4. Apnea 5. Cyanosis 6. Others symptoms________________ | |  |
| 12 | | Date of onset of symptom | _____/_____/_____date/month/year | |  |
| 13 | | Date seen at health facility- | ____/_____/_____date/month/year | |  |
| 14 | | Dose antibiotic prescribed? | 1. Yes 2. No | |  |
| 15 | | If your answer is yes for how many days prescribed | _____________________ | |  |
| 16 | | Name of antibiotic prescribed? | __________________________  ____________________________ | |  |
| 17 | | Is there any of the following complications present for you or your child | | |  |
| 17.1 | | Edema of the face? | 1. Yes, 2. No 3. Not mentioned | |  |
| 17.2 | | Sub Conjunctival Hemorrhage? | 1. Yes, 2. No 3. Not mentioned | |  |
| 17.3 | | Weight loss? | 1. Yes, 2. No 3. Not mentioned | |  |
| 17.4 | | Pneumonia? | 1. Yes, 2.No 3. Not mentioned | |  |
| 17.5 | | Seizure? | 1. Yes, 2. No 3. Not mentioned | |  |
| 17.6 | | Hernia? | 1. Yes, 2. No 3. Not mentioned | |  |
| 17.7 | | Was the participant Hospitalized? | 1. Yes, 2. No 3. Not mentioned | |  |
| 17.8 | | If yes, duration (in days) of hospitalization: | ________________ | |  |
| 18 | | Did you /your child ever been sick with pertussis infection? | 1. Yes 2. No | |  |
| 1. **Possible source of exposure and Vaccination status** | | | | | |
| 19 | | Do you have a contact with pertussis cases (a contact history with person show a cough lasting at least two weeks with at least one of the following? Inspiratory “whooping”, post-tussive vomiting (vomiting immediately after coughing and without other apparent causes, paroxysms (fits of coughing)? | | 1. Yes 2. No |  |
| 20 | | If the above question is yes: date of suspected exposure | | __ / ___ / ____ |  |
| 21 | | Is there a pertussis cases in your family? | | 1. Yes 2. No |  |
| 22 | Did you/your child vaccinate against pertussis? | | | 1. No 2. Yes 3. Unknown |  |
| 23 | | If yes; could you tell me the date of vaccination | | ________________ |  |
| 24 | | If yes how many pentavalent dose received/Vaccinated | | __________ |  |
| 25 | | If your answer is no what is the main reason | 1. The health facility is far 2. I do not know the time of vaccination day 3. The vaccine will hurt me /my child. 4. The vaccine does not prevent Pertussis 5. Other specify --------------- | |  |
| 1. **Housing condition** | | | | |  |
| 26 | | Does your house have two or more rooms? | 1. Yes 2. No | |  |
| 27 | | Does your house have one or more windows? | 1. Yes 2. No | |  |
| 28 | | If Yes, specify the direction of windows –––––––––––– |  | |  |
| 1. **Knowledge about pertussis** | | | | |  |
| 29 | | Did you know about the mode of transmission for pertussis? | 1. Yes 2. No | |  |
| 30 | | Did you know prevention and control methods of pertussis? | 1. Yes 2. No | |  |
| 31 | | Did you know about the presence of appropriate treatment for pertussis? | 1. Yes 2. No | |  |
| 32 | | Did you heard about pertussis is a vaccine-preventable disease? | 1. Yes 2. No | |  |

የትክትክ በሽታ ወረርሽኝ መረጃ መሰብሰቢያ የአማርኛ መጠይቅ

የመጠይቅ ቁጥር______________

የቀበሌ ስም______________________

የመረጃ ሰብሳቢው ስም___________________ ፊርማ_____________ ቀን _________

የተቆጣጣሪው ስም_______________________ፊርማ___________ቀን_____________________

ተሳታፊ: በሽታ የታየበት የቤተሰብ አባል ( እናት፣ አባት፣ እህት፣ ወንድም ፣ ሌሎች የቤተሰብ አባላት)

የተሳታፊ በሽታ ሁኔታ፡-ምልክት የታየበት(Case)______ ምልክት ያልታየበት(Control) ____

| 1. **አጠቃላይ መረጃ** | | | | | |
| --- | --- | --- | --- | --- | --- |
| ተ.ቁ | | ጥያቄ | መልስ | | ምርመራ |
| 1 | | ዕድሜ | __________ ዓመት | |  |
| 2 | | ጾታ | 1. ሴት 2. ወንድ | |  |
| 3 | | ሀይማኖት | 1. ኦርቶዶክስ ክርስቲያን 2. ሙስሊም 3. ፕሮቴስታንት 4. ሌላ (ይግለጹ) | |  |
| 4 | | ስራ (የበሽታ ምልክት የታየበት/የበሽታ ምልክት ያልታየበት) | 1. ለስራ ያልደረስ 2. አርሶ ኣደር 3. የቤት እመቤት 4. ተማሪ 5. የመንግስት ሰራተኛ 6. የግል 7. ሌላ(ይገለጽ)------------------- | |  |
| 5 | | የትምህርት ሁኔታ (የበሽታ ምልክት የታየበት/የበሽታ ምልክት ያልታየበት) | 1. ለትምህርት ያልደረሰ 2. ማንበብ እና መጻፍ የማይችል 3. ማንበብ እና መጻፍ የሚችል 4. እስከ 1ኛ ደረጃ ድረስ (1-8) 5. ሁለተኛ ደረጃ (9-12) 6. ኮሌጅ ዲፕሎማ እና በላይ | |  |
| 6 | | የጋብቻ ሁኔታ (የበሽተኛ ተንክባካቢ) | 1. ያገባ/ች 2. ያላገባ/ች 3. የሞተበት/ባት 4. ተለያይተው የሚኖሩ 5. አግብቶ የፈታ/የፈታች | |  |
| 7 | | የትምህርት ሁኔታ (የበሽተኛ አሳዳጊ/ተንክባካቢ) | - - - 1. ማንበብ እና መጻፍ የማይችል       2. ማንበብ እና መጻፍ የሚችል       3. እስከ 1ኛ ደረጃ ድረስ (1-8)       4. ሁለተኛ ደረጃ (9-12)       5. ኮሌጅ ዲፕሎማ እና በላይ | |  |
| 8 | | ስራ (የበሽተኛ አሳዳጊ/ተንክባካቢ) | አርሶ ኣደር  የቤት እመቤት  ተማሪ  የመንግስት ሰራተኛ  የግል  ሌላ (ይገለጽ)------------------- | |  |
| 9 | | የመኖሪያ ቦታ ከክትባት ጣቢያ ያለው እርቀት | 1. ከ 5 ኪሎ ሜትር በታች 2. ከ 5 ኪሎ ሜትር በላይ | |  |
| 10 | | የቤተሰብ አባላት ብዛት | _______________ ቁጥር | |  |
| 1. **በበሽተኛው ላይ የሚታዩ የበሽታ ምልክቶች እና ተያያዥ ችግሮች** | | | | | |
| 11 | | እርስዎ / ልጅዎ/ የታየብዎት የበሽታ ምልክት ምንድን ነው?  . | 1. ፓሮሳይሲማል ሳል 2. ከፍተኛ ተከታታይ ሳል 3. ከተከታታይ ሳል በኋላ ትውኪያ 4. ጊዜያዊ መተንፈስ ማቆም (ትንፋሽ ማጠር) 5. ሌላ ምልክት________________ | |  |
| 12 | | በሽታው ምልክት የጀመረበት ቀን | _____/_____/_____ ቀን / ወር/ ዓ ም | |  |
| 13 | | በጤና ተቋም የታዩበት ቀን | _____/_____/_____ ቀን / ወር/ ዓ ም | |  |
| 14 | | ለትክትክ የሚሆን መድኃኒት ወስደዋልን? | 1. አዎ 2. የለም | |  |
| 15 | | መልስዎ አዎ ከሆን ለስንት ቀን ወስደዋል? | ____________________የቀን ብዛት | |  |
| 16 | | የወስዱት የመድኃኒት አይነት ይጠቀስ? | __________________________  __________________________ | |  |
| 17 | | በእርስዎ ወይም በልጅዎ ላይ የተከሰቱ ውስብስብ (ተጓዳኝ) የትክትክ በሽታ ምልክቶች | | |  |
| 17.1 | | ከባድ የሳንባ ምች | 1. አዎ 2. የለም | |  |
| 17.2 | | የደም መፍሰስ | 1. አዎ 2. የለም | |  |
| 17.3 | | የክብደት መቀነስ | 1. አዎ 2. የለም | |  |
| 17.4 | | የቆዳ ቀለም መቀየር | 1. አዎ 2. የለም | |  |
| 17.5 | | እርስዎ ወይም ልጅዎ ሆስፒታል ገብቶ ነበር? | 1. አዎ 2. የለም | |  |
| 17.6 | | መልስዎ አዎ ከሆነ ሆስፒታል ተኝተው የቆዩበት ቀን ስንት ነበር | ________________ የቀን ብዛት | |  |
| 18 | | እርስዎ ወይም ልጅዎ የትክትክ በሽታ ከዚህ በፊት ታመው ያውቃሉ? | 1. አዎ 2. የለም | |  |
| 1. **የተጋላጭነት ምንጭ እና የክትባት ሁኔታ** | | | | | |
| 19 | | በትክትክ ከተያዘ ሰው ጋር ንክኪ ነበረዎት? | | 1. አዎ 2. የለም |  |
| 20 | | መልስዎ አዎ ከሆነ ንክኪ የነበረበት ቀን መቸ ነው? | | __ / ___ / ___ |  |
| 21 | እርስዎ/ልጅዎ የትክትክ ክትባት ተከትበዋል? | | | 1. አዎ 2. የለም |  |
| 22 | | መልስዎ አዎ ከሆነ ምን ያህል የፔንታቫለንት መጠን ክትባት ተከትበዋል | | 1. አንድ 2. ሁለት 3. ሶስት |  |
| 23 | | የጥያቄ ተራ ቁጥር 21 መልስ አዎ ከሆነ የመጨረሻውን ክትባት የተከተቡበት መቸ ነው? | | _____/_____/______ቀን/ወር/ ዓ ም |  |
| 24 | | የጥያቄ ተራ ቁጥር 21 መልስ የለም ከሆነ ያልተከተቡበት ምክንያት ምንድን ነው? | 1. ከትባት የሚሰጥበት ተቋም ሩቅ በመሆኑ 2. የክትባት የሚሰጥበትን ቀን አላውቅም 3. ክትባቱ እኔንም ሆነ ልጄን ይጎዳል ብየ ስለፈራሁ 4. ክትባቱ ትክትክን በሽታን መከላከል ስለማይችል 5. ሌላ (ይገለጽ) --------------- | |  |
| 1. **የመኖሪያ ቤት ሁኔታ** | | | | |  |
| 25 | | መስኮት ባለው ቤት ውስጥ ነው የሚኖሩት? | 1. አዎ 2. የለም | |  |
| 26 | | መልስዎ አዎ ከሆን መስኮቱን በየቁኑ በመክፈት ይጠቀሙበታል? | 1. አዎ 2. የለም | |  |
| 1. **ስለ ትክትክ በሽታ የግንዛቤ (እውቀት) ሁኔታዎች** | | | | |  |
| 27 | | ትክትክ በሽታ የሚተላለፍበትን መንገድ ያውቃሉ? | 1. አዎ 2. የለም | |  |
| 28 | | የትክትክ በሽታ መከላከያ ዘዴዎችን ያውቃሉ? | 1. አዎ 2. የለም | |  |
| 29 | | ለ ትክትክ በሽታ ተገቢው ህክምና ስለመኖሩ ያውቃሉ? | 1. አዎ 2. የለም | |  |
| 30 | | ትክትክ በሽታን በክትባት መከላከል እንደሚቻል ያውቃሉ? | 1. አዎ 2. የለም | |  |
